# Identification and Functional Analysis of GJA8 Mutation in a Chinese Family with Autosomal Dominant Perinuclear Cataracts

**DOI:** 10.1371/journal.pone.0059926

**Published:** 2013-03-29

**Authors:** Dongmei Su, Zhenfei Yang, Qian Li, Lina Guan, Huiling Zhang, Dandan E, Lei Zhang, Siquan Zhu, Xu Ma

**Affiliations:** 1 Department of Genetics, National Research Institute for Family Planning, Beiing, China; 2 Capital Medical University,Beijing Ophthalmology and Visual Sciences Key Lab, Beijing, China; Innsbruck Medical University, Austria

## Abstract

Congenital cataract is a clinically and genetically heterogeneous group of eye disorders that causes visual impairment and childhood blindness. The purpose of this study was to identify the genetic defect associated with autosomal dominant congenital perinuclear cataract in a Chinese family. A detailed family history and clinical data of the family were recorded, and candidate gene sequencing was performed to screen for mutation-causing disease in our study. Direct sequencing revealed a c.601G>A (p.E201K) transversion in exon 2 of GJA8. This mutation co-segregated with all affected individuals in the family and was not found in unaffected family members or 100 unrelated controls. The function and mechanism of novel GJA8 point mutation E201K in Chinese patients were then investigated in this study. We found E201K aberrantly located in cytoplasm and prevented its location in the plasma membrane. Induction of E201K expression led to a decrease in cell growth and viability by MTT (3-(4,5-Dimethylthiazol-2-yl)-2,5-diphenyltetrazolium bromide) assay. Our study provides important evidence that GJA8 is a disease-causing gene for congenital cataract and that mutation of GJA8 has a potential causative effect.

## Introduction

Congenital cataract is the most common cause of treatable childhood blindness. More than 1 million blind children in Asia suffer from cataract [1]. Genetically, autosomal dominant inheritance (ADCC) is the most common mode of transmission, and nearly a third of cases show a positive family history [2], [3]. The prevalence of congenital cataracts is 1 to 6 per 10,000 live births [4]. Approximately half are inherited, either in isolation or as part of a syndrome of ocular or systemic anomalies [2].The penetrance of congenital cataract is almost complete but expressivity is highly variable. According to morphology, congenital cataracts can be classified into several subtypes: sutural, pulverulent, whole lens, nuclear, lamellar(also referred to as perinuclear), cortical, polar, cerulean, coralliform, and other minor subtypes [5], [6]. These cataract subtypes can result from mutations at different genetic loci and can have different inheritance patterns.

To date, more than 30 independent loci and 20 cataract-related genes have been identified as being associated with isolated autosomal dominant congenital cataract (ADCC). Several genes are highly expressed in the lens and have been associated with congenital cataracts that can be considered as candidate genes for hereditary cataracts. And these genes include gap junction protein, alpha 3, *(GJA3)*, and gap junction protein, alpha 8, *(GJA8)*; crystallins such as αA-crystallin (CRYAA), βA1-crystallin (CRYBA1), βB1-crystallin (CRYBB1), βB2-crystallin (CRYBB2), γC-crystallin (CRYGC), γD-crystallin (CRYGD) and γS-crystallin (CRYGS), intrinsic member protein (LIM2), and major intrinsic protein (MIP), cytoskeletal proteins, such as beaded filament structural protein 1 (BFSP1), beaded filament structural protein 2 (BFSP2), transcription factors, such as paired-like homeodomain 3 (PITX3), heat shock transcription factor 4 (HSF4), Maf-like protein gene (MAF), and paired box gene 6 (PAX6), and others, such as chromatin modifying protein 4B (CHMP4B) and Eph-receptor type-A2 (EPHA2) and so on[7]–[12].

In this study, we applied a functional candidate approach to test the known genes in a Chinese family. A novel missense mutation in GJA8 was identified to be responsible for the cataracts in the family. Functional analysis showed that mutation of GJA8 prevented its location in the plasma membrane and led to a decrease in cell growth and proliferation. These results suggest the novel missense mutation in GJA8 may contribute to the development of nuclear cataract.

## Methods

### Family Data and Clinical Examination

The three generation Chinese cataract family was recruited from Beijing tongren hospital, capital medical University, Beijing, China. The research was approved by the ethics committee of Capital Medical University. Informed consent was obtained from all participants of the family, from Guangdong province. The study protocol followed the principles of the Declaration of Helsinki.

Detailed family medical history was recorded by interviewing the family members. All participating members underwent ophthalmic examination including visual acuity, slit lamp examination, intraocular pressure measurement, ultrasonography, fundus examination of the dilated pupil. Slit-lamp photography was performed to document the phenotype of the cataract patients. 100 unrelated subjects without cataracts were recruited from the Ophthalmology Clinic of Beijing Tongren Hospital as normal controls. They were given complete ophthalmologic examinations and did not have eye diseases except mild myopia.

### Clinical Examination and Isolation of Genomic DNA

About 2 ml of peripheral blood was collected from the family members who took part in the study. Genomic DNA was extracted from blood using the QIAamp Blood kit (Qiagen, Valencia, CA).

### Mutation Screening

We used the functional candidate gene analysis approach, 11 candidate genes, including CRYAA, CRYAB, CRYBA1, CRYBB1, CRYBB2, CRYGC, CRYGD, CRYGS, GJA3, GJA8, MIP, HSF4, and BFSP2 were analyzed. Each exon and intron-exon junction of the genes were amplified by polymerase chain reaction (PCR) with primers listed in Table 1. The PCR products of proband were sequenced using ABI 3730 Automated Sequencer (PE Biosystems, Foster City, CA). The sequencing results were analyzed using Chromas 2.33 (http://www.technelysium.com.au/chromas.html) and compared to the reference sequence in the NCBI (http://www.ncbi.nlm.nih.gov/) database.

**Table 1:** Primers used for PCR.

Name	Forward (5′–3′)	Reverse (5′–3′)
CRYAA-1	AGCAGCCTTCTTCATGAGC	CAAGACCAGAGTCCATCG
CRYAA-2	GGCAGGTGACCGAAGCATC	GAAGGCATGGTGCAGGTG
CRYAA-3	GCAGCTTCTCTGGCATGG	GGGAAGCAAAGGAAGACAGA
CRYAB-1	AACCCCTGACATCACCATTC	AAGGACTCTCCCGTCCTAGC
CRYAB-2	CCATCCCATTCCCTTACCTT	GCCTCCAAAGCTGATAGCAC
CRYAB-3	TCTCTCTGCCTCTTTCCTCA	CCTTGGAGCCCTCTAAATCA
CRYBA1–1	GGCAGAGGGAGAGCAGAGTG	CACTAGGCAGGAGAACTGGG
CRYBA1–2	AGTGAGCAGCAGAGCCAGAA	GGTCAGTCACTGCCTTATGG
CRYBA1–3	AAGCACAGAGTCAGACTGAAGT	CCCCTGTCTGAAGGGACCTG
CRYBA1–4	GTACAGCTCTACTGGGATTG	ACTGATGATAAATAGCATGAACG
CRYBA1–5	GAATGATAGCCATAGCACTAG	TACCGATACGTATGAAATCTGA
CRYBA1–6	CATCTCATACCATTGTGTTGAG	GCAAGGTCTCATGCTTGAGG
CRYBB1–1	CCCTGGCTGGGGTTGTTGA	TGCCTATCTGCCTGTCTGTTTCTC
CRYBB1–2	TAGCGGGGTAATGGAGGGTG	AGGATAAGAGTCTGGGGAGGTGG
CRYBB1–3	CCTGCACTGCTGGCTTTTATTTA	TCTCCAGAGCCCAGAACCATG
CRYBB1–4	CCAACTCCAAGGAAACAGGCATA	CCTCCCTACCCACCATCATCTC
CRYBB1–5	TAGACAGCAGTGGTCCCTGGAGA	AGCACTGGGAGACTGTGGAAGG
CRYBB1–6	CCTAGAAAAGGAAACCGAGGCC	AGCGAGGAAGTCACATCCCAGTA
CRYBB2–1	GTTTGGGGCCAGAGGGGAGTGGT	TGGGCTGGGGAGGGACTTTCAGTA
CRYBB2–2	CCTTCAGCATCCTTTGGGTTCTCT	GCAGTTCTAAAAGCTTCATCAGTC
CRYBB2–3	GTAGCCAGGATTCTGCCATAGGAA	GTGCCCTCTGGAGCATTTCATAGT
CRYBB2–4	GGCCCCCTCACCCATACTCA	CTTCCCTCCTGCCTCAACCTAATC
CRYBB2–5	CTTACCCTTGGGAAGTGGCAATGG	TCAAAGACCCACAGCAGACAAGTT
CRYGC-1	TGCATAAAATCCCCTTACCG	CCTCCCTGTAACCCACATTG
CRYGC-2	TGGTTGGACAAATTCTGGAAG	CCCACCCCATTCACTTCTTA
CRYGD-1	CAGCAGCCCTCCTGCTAT	GGGTCCTGACTTGAGGATGT
CRYGD-2	GCTTTTCTTCTCTTTTTATTTCTGG	AAGAAAGACACAAGCAAATCAGT
CRYGS-2	GAAACCATCAATAGCGTCTAAATG	TGAAAAGCGGGTAGGCTAAA
CRYGS-3	AATTAAGCCACCCAGCTCCT	GGGAGTACACAGTCCCCAGA
CRYGS-4	GACCTGCTGGTGATTTCCAT	CACTGTGGCGAGCACTGTAT
GJA3–1	CGGTGTTCATGAGCATTTTC	CTCTTCAGCTGCTCCTCCTC
GJA3–2	GAGGAGGAGCAGCTGAAGAG	AGCGGTGTGCGCATAGTAG
GJA3–3	TCGGGTTCCCACCCTACTAT	TATCTGCTGGTGGGAAGTGC
GJA8–1	CCGCGTTAGCAAAAACAGAT	CCTCCATGCGGACGTAGT
GJA8–2	GCAGATCATCTTCGTCTCCA	GGCCACAGACAACATGAACA
GJA8–3	CCACGGAGAAAACCATCTTC	GAGCGTAGGAAGGCAGTGTC
GJA8–4	TCGAGGAGAAGATCAGCACA	GGCTGCTGGCTTTGCTTAG
MIP-1	GTGAAGGGGTTAAGAGGC	GGAGTCAGGGCAATAGAG
MIP-2,3	CGGGGAAGTCTTGAGGAG	CACGCAGAAGGAAAGCAG
MIP-4	CCACTAAGG TGGCTGGAA	CTCATGCCCCAAAACTCA
HSF4–1	CATCCCATCCAGCCAGCCTTTTC	GGGCATGGGTGTTCACTGACGT
HSF4–2	CCTCGACCCATATCCCCGTAAG	GCAGGAGCAAGGCAGGCAGTC
HSF4–3	GCGGGAATGAGCAAAGAGGAGG	GCCAAGGCAGGAGAGAGGAAGG
HSF4–4	TCCCCAGCCTCGCCATTCT	CCCGGTGAAGGAGTTTCCAGAG
HSF4–5	GCTGGGGCCTGAGGGAG	GGCTTCCATCTTCTCTTCCTTTT
BFSP2 (1a)	AATGCACAAACCCAAATGGT	AGGCCCTGSSGACACT
BFSP2 (1b)	GAGAGGCGAGTGGTAGTGGA	GGCCTCAGCCTACTCACAAC
BFSP2 (2)	TGCAGACAGAGCATTTCCAC	GAGGGGTGTGAGCTGGATAA
BFSP2 (3)	GCTGCAATTGCCTTCATTTT	GGGTAACCTGACCCAACTTCA
BFSP2 (4)	TCTGTGAAGCCTGTGTCTGG	CCCGGCCTCAATTATTCTTT
BFSP2 (5)	ACCCAGGAGGAGGAGGTTGT	GGGAATCCCCTGGAAACTAA
BFSP2 (6)	GGGGAATAGTCCAGGCTACC	ATGGGTGCCTATGTGAGAGGG
BFSP2 (7)	TTGTTCCAAAGGCCAGATTC	CACTCAAGGGAATCCTTCCA

### Bioinformatics Analysis

The amino acid sequences of GJA8 from several different species were obtained from NCBI GenBank and conservation analysis was performed by CLC Main Workbench Software (Aarhus, Denmark). The function impact of the mutation was predicted by PolyPhen (http://genetics.bwh.harvard.edu/pph2/).

### Cell Culture and Transfection

The cell lines Hela were maintained in Iscove’s modified Dulbecco’s medium supplemented with 10% fetal bovine serum, 100 mg/ml penicillin and 100 mg/ml streptomycin in a humidified atmosphere containing 5% CO2 at 37°C. Transfection was carried out using Lipofectamine 2000 (Invitrogen Corporation, Carlsbad, CA, USA).

### Western Blotting

Total proteins were extracted from Hela cells transfected with GJA8-WT-GFP (wild-type), GJA8-E201K-GFP and GFP vector plasmids. After electrophoresis, a polyvinylidene fluoride membrane was incubated with anti-β-actin antibody (Sigma) and anti-GFP (Abcam). The signals were visualized by using the chemiluminescent substrate method with the SuperSignal West Pico kit provided by Pierce Co.

### Subcellular Localization

Hela cells were seeded in six-well tissue culture plates 24 h prior to transfection at approximately 60% confluency. GFP-GJA8 expression constructs containing wild-type or mutants were transfected using Lipofectamine 2000, according to the manufacturer’s instructions. Forty-eight hours after transfection, the cells were then analyzed by fluorescence microscopy.

### Cell Proliferation and MTT (3-(4,5-Dimethylthiazol-2-yl)-2,5-diphenyltetrazolium bromide) Assay

The protocol is adapted from literature methods [13], [14]. Briefly, After transfection, Iscove’s modified Dulbecco’s medium was supplemented with 5 mg/ml MTT (3-(4,5-Dimethylthiazol-2-yl)-2,5-diphenyltetrazolium bromide) solution for 4 h at 37°C. Thereafter, the medium was aspirated, and the formazan product was solubilized with 100 μl of methyl sulfoxide MTT solution was removed. Viability was assessed by measuring the absorbance at 492 nm with microplatereader.

## Results

### Clinical Evaluation

**Figure 1:**
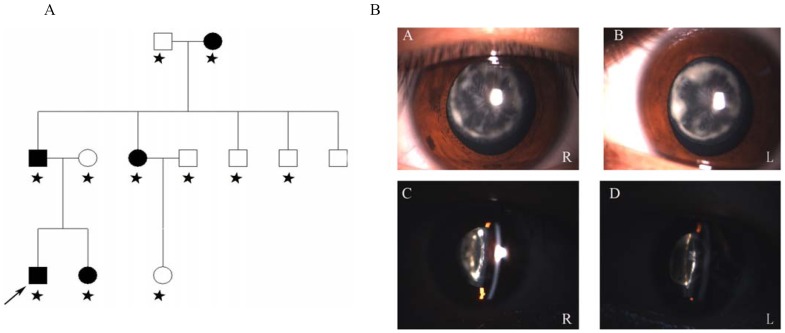
Clinical evaluation of A Chinese family with autosomal dominant cataracts. (A) A Chinese family that has had autosomal dominant cataracts. The arrow indicates the proband. Pedigree of a Chinese three-generation family with autosomal dominant cataract. The black symbols indicate individuals with a diagnosis of congenital cataract performed by genetic analysis. The arrow indicates the proband. The asterisk indicates family members who attended this study. (B) *Slit-lamp photographs of the proband.* A and C in the photograph: Slit-lamp photographs of the proband (III:1) show perinuclear cataract in the right eye. B and D in the photograph: The photographs of the proband (III:1) show perinuclear cataract involving the nucleus of the left eye.

Eleven family members of a three-generation cataract Chinese family participated in the study (five affected and six unaffected individuals) (Fig. 1A). All patients in this pedigree had bilateral cataracts. The spectrum of all patients belong to lamellar (perinuclear) cataracts, which is an subtype of congenital cataracts. Most patients experienced decreased visual acuity at 3 years of age, followed by a gradual decrease in visual acuity until surgery was required. The proband, who was a 22-year-old boy, had decreased vision at 3 years of age and was diagnosed with bilateral lamellar (perinuclear) cataract at the age of 22. Slit-lamp examination revealed opacification of perinuclear cataract with opacities involving the nucleus (Fig. 1B). His best corrected visual acuity was 0.3/0.4.

### Mutation Analysis

**Figure 2:**
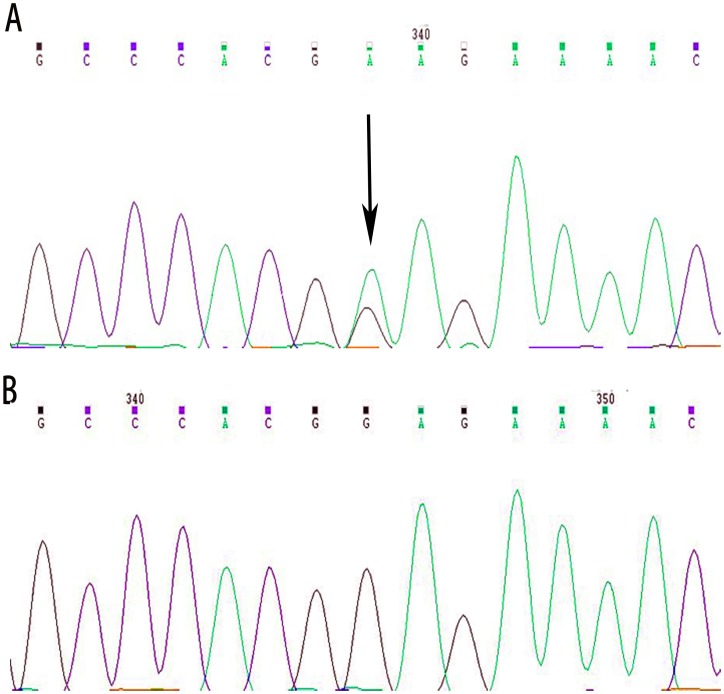
Partial sequence of GJA8 at exon2. A: Sequence of affected individual. In panel A, the heterozygous mutation c.601G>A was identified in all affected participants. B: Sequence of unaffected individual.

We identified a transversion of G>A at c.601 in exon 2 of *GJA8* in all affected individuals via direct gene sequencing of the coding regions of the candidate genes (Fig .2A). However, we did not find this mutation in any unaffected family members, or in the 100 unrelated control individuals (Fig. 2B). We found no further gene mutations in individuals from the studied family, except for a few nonpathogenic single nucleotidepolymorphisms (SNPs).

### Bioinformatics Analysis

**Figure 3:**
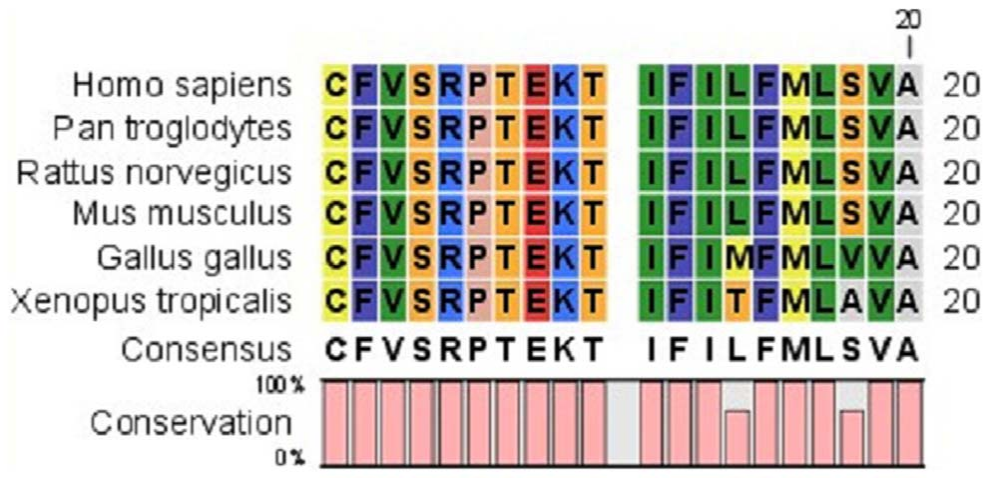
A multiple-sequence alignment of the amino acid sequence in GJA8 from different species. The alignment data indicates that the Glu at the 201st amino acid position is highly conserved among many species (indicated by an arrow).

The c.601G>A mutation resulted in a substitution of glutamic acid (Glu) with Lysine (Lys) at the 201st amino acid position (E201K). CLC Main Workbench Software revealed that the Glu at the 201st amino acid position is highly conserved among many species (Fig. 3). Furthermore, the structure and function impact of the GJA8 E201K mutation was predicted by PolyPhen (http://genetics.bwh.harvard.edu/pph2/), and the result indicated that E201K could probably be damaging, with a score of 0.999 [15], [16].

### Functional Analysis

**Figure 4:**
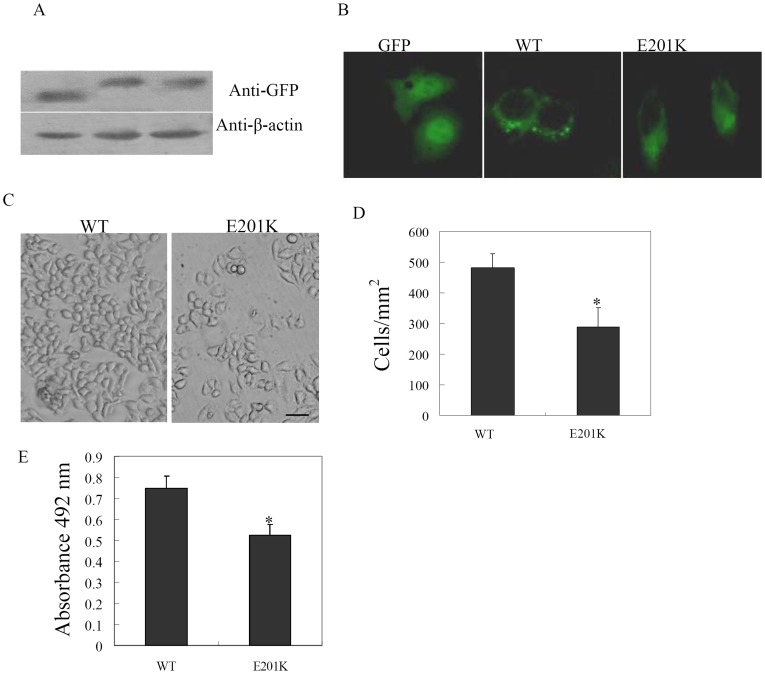
Functional analysis of GJA8 mutation. (A) Western blotting of wild-type and mutant GJA8. Hela cells were transfected with GJA8WT and GJA8E201K expression plasmids, respectively and western blotting was performed with GFP antibody. (B) Subcellular localization of GJA8. GFP-GJA8 expression constructs containing wild-type or mutant were transfected into Hela cells using Lipofectamine 2000. Localization of wild-type and mutant GJA8 GFP-fusion protein in transfected Hela cells was viewed using a fluorescent microscope. (C) Induction of GJA8E201K protein expression decreased the number of cells. Phase contrast photomicrographs from cells containing wild-type or mutant GJA8. GJA8WT (left panel) and GJA8E201K (right panel) cells were observed using a phase contrast microscope after transfection for wild-type and mutant GJA8 expression plasmids for 72 hours. Bar, 100 μm. (D) Graph shows the quantitation of these data as mean ± S.E.M. of the number of cells containing wild-type or mutant GJA8, respectively. *, p<0.05; **, p<0.01 (n = 3). (E) Induction of GJA8E201K protein expression decreased the viability of cells by MTT assay. Cells were transfected with wild-type and mutant GJA8 vectors for 3 days and cell viability was tested using MTT assay.

The amount of each of the in-vitro translated wild-type and mutant GJA8 proteins were detected by western blot analysis with anti-GFP antibody (Fig. 4A). The GJA8 WT and GJA8 E201K mutant proteins were expressed at similar levels in western blot analysis of the cell lysates, indicating that mutation did not result in the expression or instability of the protein.

We then examined the subcellular location of GJA8 wild-type and mutant protein. Subcellular localization was determined using C-terminal GFP fusion constructs of mutant and wild-type GJA8, followed by fluorescence microscopy. GFP as a control was located in the nucleus and cytoplasm and GJA8 WT was mainly detected in the plasma membrane (Fig. 4B). GJA8 E201K was aberrantly located in the cytoplasm, indicating that E201K mutation of GJA8 prevented its location in the plasma membrane.

To test directly whether expression of GJA8 mutation had a deleterious effect on cell growth or proliferation, we compared the effects of induced GJA8 WT and GJA8 E201K expression on cell numbers. As shown in Fig. 4C, a dramatic decrease in cell numbers was observed when comparing GJA8 E201K-transfected cells with GJA8 WT- transfected cells. Quantitation of these results revealed that the number of cells transfected with GJA8 E201K was only about 60% of that observed in cells transfected with GJA8 WT (Fig. 4D). Similarly, induction of GJA8 E201K expression dramatically reduced cell viability compared to GJA8 WT cells by MTT assay (Fig. 4E).

## Discussion

In this study we identified a novel G>A mutation of *GJA8* in a three-generation Chinese pedigree, which was associated with perinuclear opacities of the lens involving the nucleus. This mutation co-segregated with the phenotype and was not found in 100 unrelated control individuals.

Perinuclear cataracts affect the regions of the perinucleus, but the nucleus is clear. Perinuclear cataracts may occur in isolation or are associated with opacities involving other lens regions. However, genes associated with perinuclear cataract were not clearly identified. Mutations in *GJA8* have been considered as cataractogenesis in humans. To date, 17 mutations in this gene have been identified to contribute to assorted phenotypic cataracts [29]. In this study, we identified a novel mutation, c.601G>A in *GJA8*, which was associated with perinuclear cataract in a Chinese family.

Our study showed that GJA8WT protein was mainly detected in the plasma membrane, whereas GJA8E201K was aberrantly located in the cytoplasm. GJA8 belong to the connexin gene family [17]. Each connexin includes cytoplasmic amino and carboxyl termini as well as four transmembrane domains linked by a single cytoplasmic loop and two extracellular domains, E1 and E2 [18], [19], which are thought to play a role in connexon docking and channel gating. E201K mutation of GJA8 located in the second extracellular domains and the 201st residue is quite near the transmembrane domain. The cytoplasmic location of GJA8E201K mutant protein may be explained by the proximity of the E201K residue to the fourth transmembrane domain of GJA8.

GJA8, an important component of gap junction channels, plays an important role in intercellular communication and transfer of molecules [20]. The lens is an avascular organ dependent on gap junction-mediated transportation of ion gradients and metabolic materials and intercellular communication for organ function and homeostasis [21]–[24]. In our study, we found that induction of GJA8E201K expression dramatically reduced cell viability and proliferation compared to GJA8WT cells. At the cellular level, GJA8E201K aberrant location may lead to capacity deficiency of GJA8E201K to form functional hemichannels, and trigger a complex sequence of events including loss of membrane potential, disruption of transmembrane ion gradients, and subsequent decreased metabolic activity, and finally decreased cell growth.

Our data showed that induction of GJA8 E201K expression for 72 hours led to a decrease in the number of cells and reduction of cell viability. Recent studies have shown that gap junctional coupling provided through wild-type GJA8 had been implicated in cell proliferation. Peter et al had showed that G46V mutation of GJA8 decreased the viability of cells obtaining GJA8 G46V protein. Our in-vitro over-expression of the GJA8 E201K mutation has a similar toxic effect on cell growth as reported for other GJA8 mutations [25]–[29], however we cannot exclude that in-vivo, the effect of the p.E201K mutation might be mediated by its mislocalization in the cytoplasm, being unable to contribute to the cell metabolism in the lens.
